# Solving protein structures by combining structure prediction, molecular replacement and direct-methods-aided model completion

**DOI:** 10.1107/S2052252523010291

**Published:** 2024-01-13

**Authors:** Zengru Li, Haifu Fan, Wei Ding

**Affiliations:** a Beijing National Laboratory for Condensed Matter Physics, Institute of Physics, Chinese Academy of Sciences, Beijing 100190, People’s Republic of China; b School of Physical Sciences, University of Chinese Academy of Sciences, Beijing 100049, People’s Republic of China; Chinese Academy of Sciences, China

**Keywords:** *IPCAS*, *AlphaFold*, molecular replacement, direct-methods-aided model completion, phasing, protein structures

## Abstract

Highly accurate protein structure prediction can generate accurate models of protein and protein–protein complexes in X-ray crystallography. However, the question of how to make more effective use of predicted models for completing structure analysis, and which strategies should be employed for the more challenging cases such as multi-helical structures, multimeric structures and extremely large structures, both in the model preparation and in the completion steps, remains open for discussion. In this paper, a new strategy is proposed based on the framework of direct methods and dual-space iteration, which can greatly simplify the pre-processing steps of predicted models both in normal and in challenging cases. Following this strategy, full-length models or the conservative structural domains could be used directly as the starting model, and the phase error and the model bias between the starting model and the real structure would be modified in the direct-methods-based dual-space iteration. Many challenging cases (from CASP14) have been tested for the general applicability of this constructive strategy, and almost complete models have been generated with reasonable statistics. The hybrid strategy therefore provides a meaningful scheme for X-ray structure determination using a predicted model as the starting point.

## Abstract 2

We propose a direct-methods-based dual-space iteration strategy for model completion of molecular replacement (MR) with predicted models. Direct methods has been demonstrated as a powerful tool for phase optimization in protein crystallography, whereas the dual-space iterative strategy is particularly suitable for solving crystallographic protein-complex structures starting from a small subunit. This combined approach provides a shortcut in simplifying the pre-processing steps of predicted models for MR and for final model completion.

## 1. Introduction

X-ray crystallography is the primary method for resolving the structure of macromolecules, and the phase problem is the core issue in this field. With the development of protein structure prediction, the molecular replacement (MR) method, which is based on the use of similar models for initial phase calculation, has further increased its priority. The work of McCoy *et al.* (2022 ▸) explored the prospects for changes in phasing methods, and in particular the prospects for MR phasing using *in silico* models. Similar works are available (Baek *et al.*, 2021 ▸; Pereira *et al.*, 2021 ▸; Medina *et al.*, 2022 ▸; Simpkin *et al.*, 2022 ▸; Terwilliger *et al.*, 2022 ▸) and a general point arises that, with continuous improvement of prediction accuracy, the focus has shifted to corrections for model bias. This can mainly be divided into two aspects: treatment of predicted models and reduction of the electron density map bias introduced by the model. For the treatment of predicted models, a common approach is to adjust the model based on the predicted error. *Phenix* trims *AlphaFold* models into domains based on plDDT to dock in the map (Terwilliger *et al.*, 2022 ▸). *Slice’N’Dice* slices the model into distinct structural units by removing low-confidence regions and converts the per-residue quality scores into predicted *B* factors (Simpkin *et al.*, 2022 ▸). *ARCIMBOLDO_SHREDDER* also removes low-confidence regions and decomposes the units using *ALEPH* (Medina *et al.*, 2022 ▸). *AMPLE* truncates inaccurate predicted regions in the model based on local RMS error estimates in the *B* factor column of the model (Pereira *et al.*, 2021 ▸). Various methods also have been developed to reduce the electron density map bias introduced by the model. These include the estimation of *SIGMAA* for model phases (Read, 1986 ▸, 1997 ▸), the calculation of composite omit maps (Hodel *et al.*, 1992 ▸), density modification methods with desirable phase combinations (Cowtan, 1999 ▸) and the prime-and-switch method (Terwilliger, 2004 ▸). It is obvious that both of these approaches are effective solutions; however, the direct methods and dual-space iterative strategy mentioned later provides a new shortcut for the process of predicted models and the final refinement from the perspective of phase and model iterative optimization.

Our previous research has demonstrated the effectiveness of the phase optimization method using direct methods (Fan & Gu, 1985 ▸; He *et al.*, 2007 ▸; Zhang *et al.*, 2015 ▸; Fan *et al.*, 2014 ▸; Zeng *et al.*, 2018 ▸, 2020 ▸). The dual-space iteration strategy, which is based on direct methods, can be employed for phase extension and model completion. Numerous test cases have demonstrated the ability of the method to produce final structures with high completeness. In particularly challenging cases where model completeness is between 30 and 50%, phase errors are over 70°, or the resolution ranges from 4 to 5 Å, the method still exhibits impressive performance (Fan *et al.*, 2014 ▸). Aforementioned characteristics suggest potential applications of this method in the combination of structure prediction and experiment. Specifically, in cases where the MR method produces ideal statistical results, the MR model can be directly refined to achieve high-precision three-dimensional crystal structures. Alternatively, conservative structural domains can be selected as search models to reduce model completeness and improve accuracy, facilitating the success of MR. Subsequently, direct-methods-aided model completion can be employed to refine the completeness and accuracy of the results, leading to high-precision three-dimensional crystal structures.

The use of predicted models as search models for MR has been submitted in several publications (Kryshtafovych, Moult *et al.*, 2021 ▸; Pereira *et al.*, 2021 ▸; McCoy *et al.*, 2022 ▸; Simpkin *et al.*, 2022 ▸), yet the subsequent refinement of the model based on direct methods is reported for the first time. Specifically, we tested different combinations of predicted models by *AlphaFold* (Jumper *et al.*, 2021 ▸) from CASP14 (Kryshtafovych, Schwede *et al.*, 2021 ▸) in three cases: full-length model, multiple single-domain models and individual single-domain model. In this strategy, we performed MR using *Phaser* (McCoy *et al.*, 2007 ▸) in *CCP4* (Winn *et al.*, 2011 ▸), followed by phases extension using *OASIS* (Zhang *et al.*, 2010 ▸), density modification using *DM* (Cowtan, 1994 ▸) or *Parrot* (Cowtan, 2010 ▸), and alternative model building using *Phenix.AutoBuild* (Terwilliger *et al.*, 2008 ▸) and *Buccaneer* (Cowtan, 2006 ▸) within the framework of *IPCAS 2.0* (Ding *et al.*, 2020 ▸). Our results demonstrate that our approach effectively corrects model bias introduced by the predicted models and improves the final structures.

## 2. Methods

### 2.1. Test data

The test cases were selected from the CASP14 website (https://predictioncenter.org/casp14/index.cgi) and mostly represent a particular type of crystal structure, namely those that have a single protein sequence in the asymmetric unit (AU) and consist of one or a few domains where the domain is unrelated, or poorly related, to known structures, making them challenging for MR. In total, 13 crystal datasets corresponding to a total of 43 predicted models were chosen based on different modeling difficulty, including free modeling (FM), hard template-based modeling (TBM-hard), and the boundary between FM and TBM (FM/TBM). As shown in Table 1 ▸, the resolution ranged from 1.5 to 3.5 Å, and the number of residues of the model deposited in the PDB ranged from 133 to 4332. The maximum number of copies in the AU was six, and there were two cases of hetero-oligomers. Also note that each dataset includes one full-length model and one or two single-domain models. For example, in the cases of crystal 5 and crystal 10, there are five predicted models for each crystal, including two full-length models for each unique chain, two single-domain models and one multimer model. Furthermore, these datasets include some more challenging cases, such as a multi-helical structure in crystal 1, an extremely large structure in crystal 2, and multimeric structures in crystal 5 and crystal 10.

### 2.2. Model prediction

The predicted models were generated by *AlphaFold* based on the sequence files. The predicted models primarily consisted of three types, including single-domain models, full-length models and multimer models. CASP14 provides both single-domain models, which were decomposed into evaluation units (EUs) and full-length models, along with global distance test total scores (GDT_TS). We selected the predicted model with the highest GDT_TS, as higher values indicate a more accurate backbone and better overall model quality. All models were generated using *AlphaFold* (group No. 427). For the special case T1044 and multimer models, the corresponding *AlphaFold* models were not available on the website, so the predictions were performed using the local installation on a workstation of the code distributed via the repository at https://github.com/deepmind/alphafold. For the locally predicted models, we selected the top-ranked models. All predicted models were unmodified. The RMSD of C^α^ after alignment with the PDB model was calculated.

### 2.3. Molecular replacement

Molecular replacement was performed by auto mode in *Phaser*. We tested three scenarios with different search models, including a full-length model, multiple single-domain models and an individual single-domain model. When using a full-length model or individual single-domain model, only one predicted model was used as an ensemble in *Phaser*. While using multiple single-domain models, multiple predicted single-domain models were employed as ensembles to increase the chances of success in MR. The structures generated by the program were used as the starting point for further model completion, and the translation function *Z* score (TFZ) was recorded. *R* factors were calculated using *Phenix.Refine* (Afonine *et al.*, 2012 ▸) and the RMSD of C^α^ after alignment with the PDB model was calculated and recorded.

In addition, model-map CC validation was also used for model copy screening in some difficult cases to remove the unfitted parts of the MR model. Here, CC is the correlation coefficient between the model and density map, calculated by *Phenix.get_cc_mtz_pdb* when it is lower than 0.6, this means that there is an unsatisfactory match between the structure and the electron density, and the model can be deleted.

### 2.4. Phase extension and model completion

Further phase extension and model completion were implemented in the direct-methods-aided dual-space iterative phasing and model-building workflow in *IPCAS 2.0*. The workflow can be divided into four parts: (1) Reciprocal-space phase refinement by *OASIS*. The initial model and phases produced by MR and restrained refinement will be delivered to the direct-methods-aided software *OASIS* for phase refinement and extension. (2) non-crystallographic symmetry (NCS) matrices searching by *Phenix.Find_NCS_From_Density* (Terwilliger, 2013 ▸). If there are more than one copy of the molecule in the AU, multifold operator searching will be performed by *Phenix.Find_NCS_ From_Density*. If the NCS operator can be found and the correlation coefficient (NCS_CC) of electron density of the related areas is greater than a certain value (0.5 for the first cycle or the largest value during the cycle), the information for the NCS matrices will be recorded. (3) Density modification by *DM* or *Parrot*. The electron density map calculated in step (1) will be further modified by *DM* or *Parrot* with the NCS information in step (2). A new MTZ file with a set of improved phases and figures of merit (FOM) will be created. (4) Real-space model building and refinement by *Buccaneer* and *Phenix.AutoBuild* in alternate mode. Many test cases show that the alternate running of *Phenix.AutoBuild* and *Buccaneer* can better prevent the process diverging or converging to one of the local extrema.

**Figure 1:**
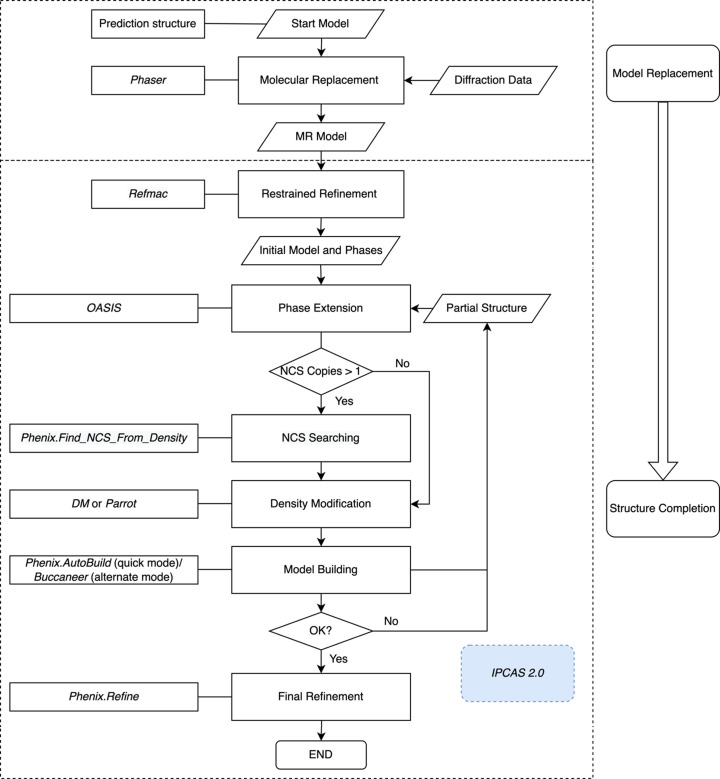
Flowchart of the combination of structure prediction, MR and direct-methods-aided model completion. Programs involved: MR, *Phaser*; direct-methods-aided model completion, *IPCAS 2.0*. Flowchart of the direct-methods-aided model completion in *IPCAS 2.0*. Programs involved: rigid-body refinement, *Refmac*; NCS searching, *Phenix.Find_NCS_From_Density*; direct-methods phasing, *OASIS*; density modification, *DM* or *Parrot*; model building, *Phenix.AutoBuild* or *Buccaneer*; final refinement, *Phenix.Refine*.

The whole procedure can be performed iteratively. During each iterative cycle, NCS matrices are updated sustainably in step (2), and the *R* factors and the modeled residues are used to monitor the result model, the result from the trial with the most modeled residues or the smallest *R* factor will be passed on to the next cycle until a satisfactory model is obtained or the maximal running cycles condition has been reached. The whole workflow is shown in Fig. 1 ▸.

By default, 50 cycles of the *OASIS*–*DM*/*Parrot*–*Phenix.AutoBuild*&*Buccaneer* iteration are performed, but are stopped halfway when *R*_free_ reaches 0.30. The best model will be further improved by *Phenix.Refine* to obtain the final structure. *R* factors were calculated using *Phenix.Refine*, and the RMSD of C^α^ after alignment with the PDB model was calculated and recorded.

## 3. Results

All calculations presented in this paper were performed on an iMac Pro (2020) (Satellite 5200–701) 3 GHz, ten-core Inter Xeon W CPU, 8GB RAM. The versions of the supported programs are *CCP4* (version 7.1.018), *Phenix* (version 1.20.1-4487-000) and *Buccaneer* (version 1.6.5). A total of 38 cases, corresponding to 13 PDB datasets, were tested using 43 predicted models.

The quality of the MR models was assessed based on the TFZ and *R* factors, whereas the quality of the *IPCAS* models was evaluated based on the completeness and *R* factors. The number of residues and RMSD of C^α^ after alignment with the PDB model were used for comparison at each step. The results starting from full-length predicted models, multiple single-domain models and individual single-domain models are listed in Tables 2 ▸, 3 ▸ and 4, respectively.

### 3.1. Results of the full-length models

The full-length predicted models can provide a general idea of the overall structure of unknown proteins, but inevitably there are some significant local deviations, especially in disordered or flexible regions.

**Figure 2:**
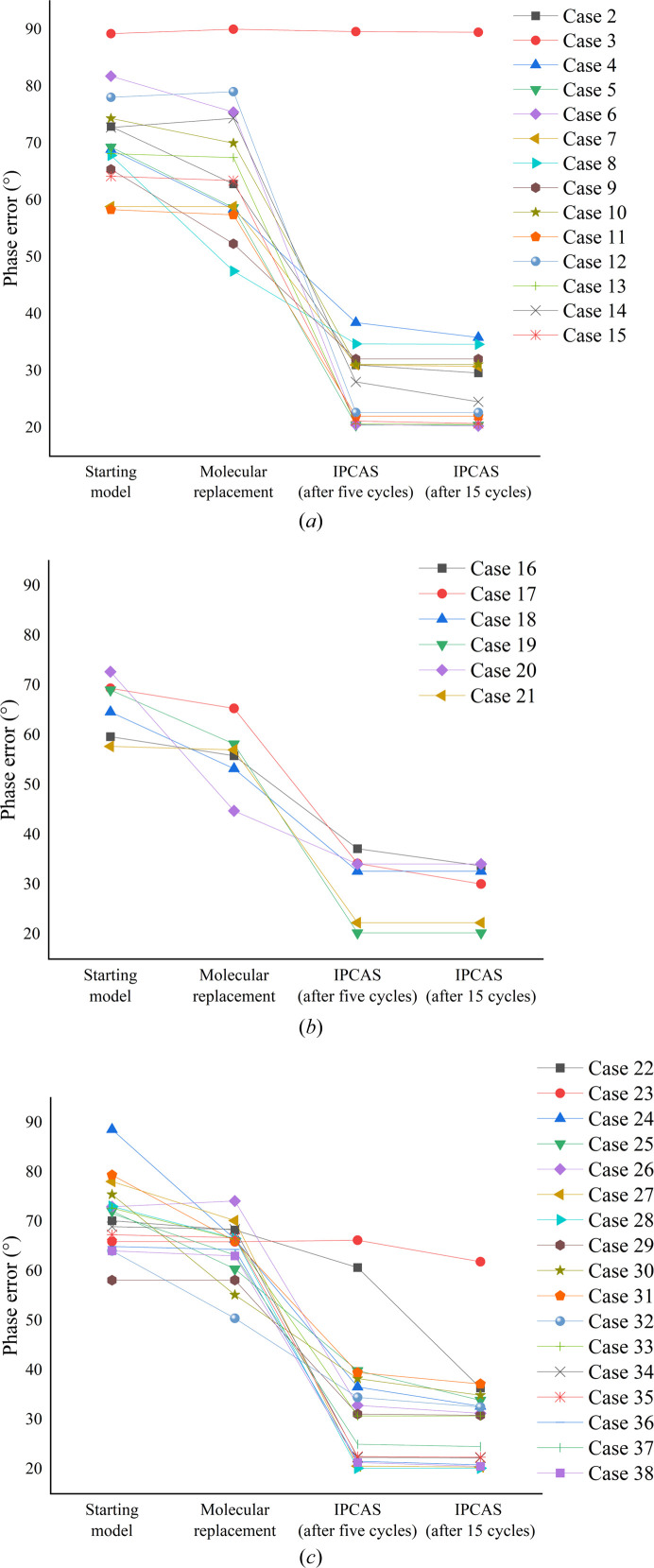
Plots of the mean phase error calculated against the crystal structure at key steps in the process. (*a*) Results of full-length models (case 3 is unsolved). (*b*) Results of multiple single-domain models. (*c*) Results of individual single-domain models (case 23 required 35 cycles of convergence).

In total, 13 out of 15 cases, with the exceptions of crystal 1 (PDB entry 6poo, case 1) and crystal 3 (PDB entry 6n64, case 3), were successfully placed by MR using the *AlphaFold* full-length models, and could be solved straightforwardly using the default protocol. As illustrated in Fig. 2 ▸(*a*), the MR method resulted in significant phase improvement, with the phase error of most cases reduced from around 90° to 48–79°. When the MR models were delivered to *IPCAS*, the local deviations and model bias of the structures could be further corrected in the direct-methods-aided model completion protocol. A significant decrease in phase error (−20 to −40°) was exhibited in the initial five cycles in *IPCAS*. Subsequent cycles, on the other hand, resemble a more refined fine-tuning process towards achieving the final structure. Eventually, *IPCAS* could build more than 90% of the completeness after 15 cycles of iteration and yield final models for most cases with acceptable refinement statistics (*R* factor ≤ 0.30, except in cases 1 and 3).

Crystals 1 and 3 possess certain structural specificity, making it difficult to find a valid solution by MR starting from the full-length model. Crystal 1 is an multi-helical structure (PDB entry 6poo). The full structure was designated the ‘multidom’ CASP target with two domains. The full-length prediction structure T1030 could not be placed by MR in case 1. Crystal 3 has six copies of the sequence in the AU in three dimers. Although the full-length prediction structure T1032 could be placed by MR in case 3, it resulted in very high *R* factors (>0.55) which posed a significant challenge for the subsequent model completion.

Also note that two PDB structures, crystal 5 (PDB entry 6px4) and crystal 10 (PDB entry 7m5f), each containing two unique chains, were tested using two different strategies. First, the prediction structures of these two unique chains were used as distinct components in MR, such as cases 5 and 11. Second, the multimer predicted model was treated as one component, such as cases 6 and 12.

### 3.2. Results of the multiple single-domain models

When the full-length model fails, trimming out unstable parts of the predicted model, such as flexible loops, and performing MR simultaneously on multiple domain models have shown to improve the success rate of MR. Six cases, cases 16–21, have been tested and all of them were successfully solved using the default protocol. As shown in Fig. 2 ▸(*b*), the phase error variation follows a similar pattern to that of the full-length cases. The optimization of phase error primarily occurs during the MR step and the first five cycles of *IPCAS* iteration. Note that, compared with the full-length models, the average phase error is much lower after the MR step (55.7° versus 65.4°). Eventually, *IPCAS* is able to reconstruct more than 93% of the completeness after 15 cycles of iteration and generate final models for all cases with acceptable refinement statistics (*R* factor ≤ 0.30).

Notably, three cases stand out: crystal 1 (PDB entry 6poo, case 16), crystal 2 (PDB entry 6vr4, case 17) and crystal 4 (PDB entry 6ya2, case 18). We failed to solve crystal 1 by MR using the full-length predicted model T1030 in case 1. On the contrary, in case 16, successful MR was achieved using the two domain models, T1030-D1 and T1030-D2. Crystal 2 corresponds to the polymerase structure. In case 17, during MR, only 8 of the 18 targets (9 predicted models with 2 copies each) could be accurately placed. Crystal 4 contains 3 copies of the sequence in the AU and has two domains corresponding to the structures of T1038-D1 and T1038-D2. In case 18, 5 out of 6 targets were accurately placed by MR.

### 3.3. Results of the individual single-domain model

Using only the single-domain portion as the starting model, a more conservative region can be selected, which effectively reduces the model bias between the predicted model and the ultimate structure, and it helps with the MR search to some extent. However, using a small model as a starting point can result in a significant loss of structural information, which can make it challenging to complete the entire structure. After the single-domain model was located and the NCS was expanded by MR, the missing regions could be further expanded through the direct-methods-aided model completion strategy in *IPCAS*. In our test cases, all 17 cases from case 22 to case 38 were solved straightforwardly with the default protocol as depicted in Fig. 1 ▸. As shown in Fig. 2 ▸(*c*), the phase error variation follows a similar pattern to that of the full-length or multiple single-domain cases. But compared with the above cases, the correction of phase error is much more significant in the last 10 cycles of *IPCAS* (2.96° versus 0.6° versus 1.3°). In addition, for case 23, the phase error is still far from convergency after 15 cycles of *IPCAS* optimization. Eventually, *IPCAS* is able to reconstruct more than 97% of the completeness after 35 cycles of iteration and generate final models for all cases with acceptable refinement statistics (*R* factor ≤ 0.30).

There are three cases worth mentioning: crystal 3 (PDB entry 6n64, case 24), crystal 4 (PDB entry 6ya2, cases 25 and 26) and crystal 5 (PDB entry 6px4, cases 27 and 28).

Crystal 3 could not be solved using the full-length prediction structure T1032 in case 3 because of the inaccuracies in MR. However, the domain model T1032-D1 could be placed unambiguously in MR in case 24, despite with high *R* factors. Crystal 4 has three copies and two domains corresponding to T1038-D1 and T1038-D2. In case 25, starting from T1038-D1, the structure could be solved straightforwardly with the default protocol. But when starting for T1038-D2 in case 26, only two of the three targets could be accurately placed by MR and the misaligned model was then deleted by CC validation (CC < 0.6) before model extension. Crystal 5 contains two unique chains and two NCS copies in the AU. Two single-domain predicted models, T1046s1-D1 and T1046s2-D1, corresponding to the smaller and larger subunits, respectively, were used as starting points in cases 27 and 28. In both cases, two copies were placed unambiguously in MR.

### 3.4. Details of remarkable cases

Crystal 1 is a multi-helical structure, which can cause modulation of the crystal diffraction data, making MR challenging, as well as difficulties in accurately predicting the interhelical angles. Crystal 2 is an extremely large structure, containing 4332 residues in the AU, which causes difficulties in MR and structure prediction. Crystal 3 has six copies, and the medium resolution and high RMSD of the predicted model make the case difficult. Crystal 4 has two domains and crystal 5 has two unique chains, both of them are multimeric structures. Details are given below.

#### 3.4.1. Crystal 1 (target T1030)

Crystal 1 (PDB entry 6poo) is a novel and predominantly helical structure, consisting of three antiparallel α-helical-bundle motifs. It is unique and belongs to a new class of gram-positive surface adhesins. The helices are arranged in four antiparallel three-helix-bundle-motif repeats, with one long helix extending into the next bundle. The highest resolution of the diffraction data is 3.03 Å.

T1030 is a predicted model of 6poo with an RMSD of 4.1 Å over 273 residues, which is a helical bundle classified as ‘multidom’ with two domains. For domain one (T1030-D1), the C^α^ RMSD was 3.1 Å over 154 residues, and for domain two (T1030-D2) the C^α^ RMSD was 2.2 Å over 119 residues.

Due to the difficulty in accurately predicting the subtle bends and kicks in the helical secondary structure, and the modulations in the diffraction data induced by a coiled coil, MR using the full-length prediction T1030 failed in case 1. In another study (Pereira *et al.*, 2021 ▸), the full-length T1030 structure was truncated to a sufficiently accurate substructure in order to achieve success in MR. But it is always difficult to find a universal truncation strategy for different structures. Using the function domain as the individual component in MR may be a better choice. According to McCoy *et al.* (2022 ▸), T1030-D2 could be placed unambiguously by MR, but the best placed model for T1030-D1 was only able to superimpose a portion of the fragment, and *R*_free_ was greater than 0.50. In our tests, cases 16, 22 and 23, the MR model starting from D1 + D2, D1 and D2 were further improved through direct-methods-aided model completion in *IPCAS*. In Fig. 2 ▸, the results show that starting from D1 + D2, case 16 exhibited the most ideal convergence speed. After MR, the phase error was reduced to approximately 56°. Furthermore, in the first five cycles of the *IPCAS* iteration, this value decreased even further to 37°. On the other hand, for the D1 model (case 22), a significant decrease in phase error occurred in the last 10 cycles of the *IPCAS* iteration.

Case 23, however, displayed a unique behavior. After MR of the D2 model, the phase error decreased to 66°. Surprisingly, after five cycles of *IPCAS* iteration, this value actually increased. It was not until the 25th cycle that the phase error started to decrease significantly, ultimately achieving convergence by the 35th cycle. Finally, in all three cases, *R* factors were below 0.30, the completeness exceeded 97%, and the RMSD of C^α^ between *IPCAS* structures and the reference structure of 6poo was less than 1.0 Å. These findings illustrate the varying convergence patterns and behaviors of different models during the *IPCAS* iteration process.

#### 3.4.2. Crystal 2 (targets T1031, T1033, T1035, T1037 and T1039–T1044)

Crystal 2 (PDB entry 6vr4) is the virion-packaged DNA-dependent RNA polymerase of crAss-like phage phi14:2 at 3.5 Å resolution. The AU contains two copies of the monomer related by a non-crystallographic twofold axis, and the entire structure comprises 4332 residues. The full polypeptide sequence is divided into nine separate domains which refer to T1031, T1033, T1035, T1037, T1039, T1040, T1041, T1042 and T1043 in CASP14, with residue numbers ranging from 95 to 404. Out of the nine separate domains, eight were classified as FM and one was classified as FM/TBM. The extremely large structure posed challenges for both structure prediction and MR, while the moderate resolution further increased the difficulty of MR.

In the study by McCoy *et al.* (2022 ▸), the authors claimed that, due to low resolution, a model required for MR had to represent, at least to some extent, the fold of the target protein. Obviously, for this special case, it is not sufficient to build the complete structure from a single target. But even starting from nine separate domains provided by CASP14, only 12 out of 18 monomeric domains could be placed in sequence using NCS relationships and refinement methods. The final structure had an RMSD over 2.5 Å when compared with the PDB structure.

**Figure 3:**
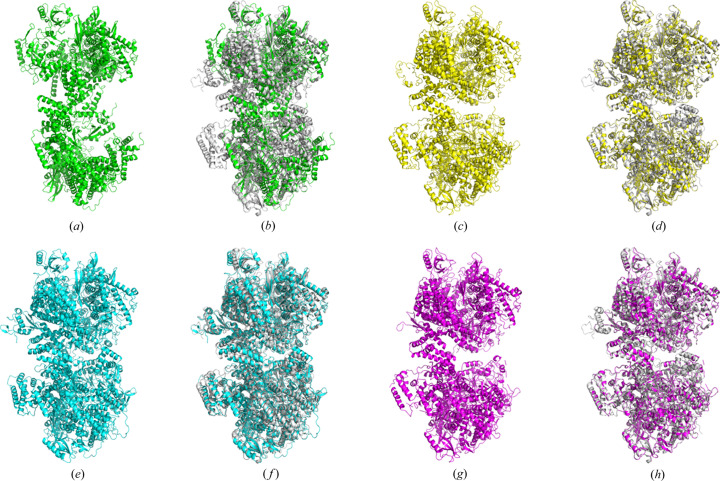
Crystal 2 (PDB entry 6vr4), targets T1031, T1033, T1035, T1037 and T1039–T1044. (*a*) Model from *Phaser* based on T1031, T1033, T1035, T1037 and T1039–T1043. (*b*) Model (*a*) superimposed with the crystal structure. (*c*) Model from *IPCAS* based on model (*a*). (*d*) Model (*c*) superimposed with the crystal structure. (*e*) Model from *Phaser* based on T1044. (*f*) Model (*e*) superimposed with the crystal structure. (*g*) Model from *IPCAS* based on model (*e*). (*h*) Model (*g*) superimposed with the crystal structure.

We also performed MR on the multiple domain models in case 17. In automatic mode, *Phaser* was just able to align 8 out of the 18 copies, including T1031, T1033, T1042 and T1043 from one copy; and T1037 and T1041 from two copies, with a C^α^ RMSD of 49.8 Å over 3204 residues. As shown in Fig. 3 ▸(*b*), the MR model is far from the final result. But the numerous errors and gaps could be largely corrected using the standard workflow of direct-methods-aided model completion in *IPCAS*. In the beginning of the third cycle, the missing parts and model bias are rapidly reconstructed and corrected. Additionally, the phase errors tend to converge starting from the fifth cycle. Finally, after a 15 cycle iteration, the errors were essentially rectified, and the gaps improved. The *IPCAS* structure exhibited excellent parameters with *R* factors of 0.21 and 0.26, completeness of 93.51%, and an RMSD of 2.4 Å over 4018 residues [as shown in Fig. 3 ▸(*c*)].

For the full-length prediction of 6vr4, the corresponding *AlphaFold* models were not available on CASP14, so the prediction was performed using the local installation. The top-ranked model was subjected to the standard procedure in case 2 (as shown in Fig. 1 ▸). The resulting final structure showed a significant improvement, with the RMSD reduced from 3.1 Å over 2166 residues to 0.5 Å over 3850 residues. The completeness also increased to 91.27%, and the *R* factors were 0.22 and 0.27, respectively [as shown in Fig. 3 ▸(*g*)].

#### 3.4.3. Crystal 3 (target T1032)

Crystal 3 (PDB entry 6n64) is the crystal structure of mouse SMCHD1 hinge domain at 3.3 Å resolution. There are six copies of the sequence in the AU in three dimers with 1071 residues. T1032 represents the predicted full-length model for this structure as published in CASP14. Compared with the PDB structure, T1032 has a long α-helix in the N-terminal which is absent in the experimental data. T1032-D1 is segmented from T1032, which corresponds to the rest of the experimentally present parts.

The predicted model had low confidence with T1032 and T1032-D1, as shown by the high RMSD values of 6.0 Å over 173 residues and 5.7 Å over 170 residues, respectively. Due to the absence of a long α-helix corresponding to the diffraction data, MR was challenging for T1032. McCoy *et al.* (2022 ▸) introduced two different approaches to finding the ideal MR solution. Both should modify the search model to eliminate the predicted deviation between model and target before MR. Despite truncation of the model, the moderate resolution and the AU with six copies also make T1032 a failed case for *AMPLE* (Pereira *et al.*, 2021 ▸).

We conducted separate tests using the full-length model T1032 and the single-domain model T1032-D1 as starting models through the standard procedure in cases 3 and 24 (as shown in Fig. 1 ▸). Without truncation, the MR with T1032 failed to position the model correctly, and the RMSD is as high as 39.0 Å. This issue was resolved using the single-domain model T1032-D1. In this model, the flexible helix present in T1032 was removed while preserving the conservation domain. Six copies of T1032-D1 were unambiguously placed by MR, despite significant deviations in the model. The resulting MR model had an RMSD of 7.4 Å over 1020 residues, which were subsequently resolved by the completion process carried out by *IPCAS*. The final model exhibited an improved RMSD of 1.2 Å over 1035 residues, with *R* factors of 0.24 and 0.25.

#### 3.4.4. Crystal 4 (target T1038)

Crystal 4 (PDB entry 6ya2) is the crystal structure of TSWV glycoprotein N ectodomain. There are three copies of the monomer in the AU, containing 551 residues. The unique chain was divided into two domains. T1038-D1 is bigger with six longer β-sheets and two α-helices, and T1038-D2 is smaller with seven shorter β-sheets and one short helix.

The first ranked *AlphaFold* model for T1038 showed a C^α^ RMSD of 2.4 Å over 190 residues when compared with the reference PDB structure. When considering the individual domains, the C^α^ RMSD was 2.5 Å over 114 residues for D1 and 1.9 Å over 76 residues for D2. Note, there are multiple β-sheets in the D2 domain.

In our tests, T1038, D1 + D2, D1 and D2 were used as MR search models separately in cases 4, 18, 25 and 26. Starting from T1038 or T1038-D1, three copies of the starting model were correctly located in MR, and the RMSDs are 2.1 Å over 551 residues and 2.1 Å over 324 residues. But for T1038-D2, although three copies of the starting model were located, one had a significantly lower CC score (<0.6) and was subsequently removed by CC validation. The same situation was found in subsequent work, when using two domains (D1 + D2) as the starting model in MR, all three copies could be identified, but one copy of D2 remained incorrect, resulting in a high RMSD of 10.7 Å over 551 residues. The incorrect placement of the D2 model directly resulted in a high phase error (74°) of the MR structure in case 26. However, due to the unique NCS search function in *IPCAS*, the accurate NCS matrix was successfully obtained in the first cycle of the *IPCAS* iteration (Ding *et al.*, 2020 ▸), and the missing part of model was completed in the third cycle, reducing the phase error to approximately 25°. The remaining three cases have a more ideal starting structure, so the correction in *IPCAS* also works well. Ultimately, in all four cases, the *R* factors were less than 0.30, the completeness was greater than 96% and the RMSD was less than 1.5 Å.

#### 3.4.5. Crystal 5 (target T1046)

Crystal 5 (PDB entry 6px4) is the crystal structure of the complex between periplasmic domains of antiholin RI and holin T from T4 phage, in H32. There are two copies of the dimer in the AU related by a non-crystallographic twofold axis, containing 427 residues. This is a typical multimeric structure; therefore, in addition to the three sets of testing schemes mentioned above (full-length, multiple single-domain, single-domain only), we also conducted a structural analysis test on the whole multimer model.

The full polypeptide sequence of antiholin RI and holin T consists of 74 and 142 residues, respectively, which correspond to targets T1046s1 and T1046s2. T1046s1 represents one chain with fewer residues, consisting of three helices. T1046s2 represents the other chain with more residues, consisting of three α-helices and five β-sheets. Each chain has one domain, denoted ‘-D1’, shown as T1046s1, T1046s1-D1, T1046s2 and T1046s2-D1. Since the corresponding *AlphaFold* multimer model was not available on the website, the prediction was made by a local installation. Compared with the reference structure, the predicted multimer model has accurate distances between subunits, with C^α^ RMSD values less than 1.2 Å over 214 residues.

**Figure 4:**
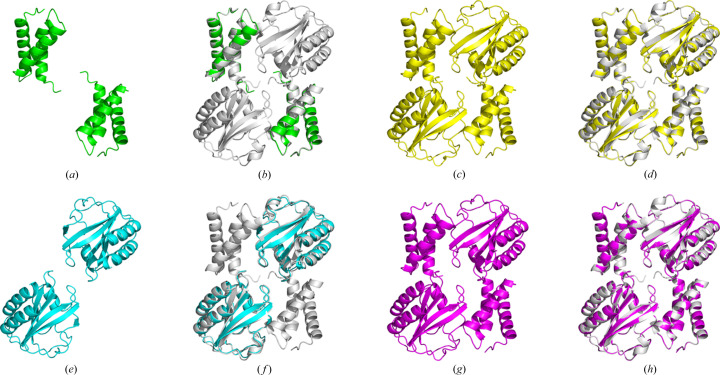
Crystal 5 (PDB entry 6px4), target T1046. (*a*) Model from *Phaser* based on T1046s1-D1. (*b*) Model (*a*) superimposed with the crystal structure. (*c*) Model from *IPCAS* based on (*a*). (*d*) Model (*c*) superimposed with the crystal structure. (*e*) Model from *Phaser* based on T1046s2-D1. (*f*) Model (*e*) superimposed with the crystal structure. (*g*) Model from *IPCAS* based on (*e*). (*h*) Model (*g*) superimposed with the crystal structure.

After MR, the predicted models were further refined using direct methods to improve the structure details. For all five cases, the final models had *R* factors no greater than 0.25, completeness greater than 98% and RMSDs less than 0.15 Å. Interestingly, in cases 27 and 28, it was observed that both the larger and the smaller domains of the single-domain models T1046s1-D1 or T1046s2-D1 could be extended to form the complete structure during the *IPCAS* iteration, as depicted in Fig. 4 ▸. Furthermore, in the case of full length and multimer structures (cases 5 and 6), the *IPCAS* models demonstrated a significant decrease in RMSD values to 0.09 Å, indicating the validity and effectiveness of the improvements made.

## 4. Discussion

The test results suggest that the direct-methods-aided dual-space iteration pipeline, in combination with the proposal strategy, can gradually reduce the deviation of the predicted model and effectively improve the completeness of the MR model. Additionally, there are still some aspects that are worthy of further discussion.

### 4.1. The characteristics of three kinds of predicted models

The full-length predicted models can provide a general idea of the overall structure of unknown proteins. When target proteins have moderate length (residues number range from 100 to 1000) and relatively conservative structure (RMSD less than 5 Å), the full-length predicted model is an ideal starting structure for MR and model completion, as shown in Table 2 ▸.

Starting from the multiple single-domain models indeed has a high success rate and good universality in various test cases. However, the key to success or failure lies in how to divide the domain appropriately. If the domain selection is too strict, it may lead to a reduction in the completeness of the model, which is not conducive to the accurate solution of the MR method. On the other hand, if the domain selection is too loose, it may introduce flexible regions, which will also pose difficulties in the subsequent MR solution. To address this challenge, it is crucial to strike a balance in domain selection. One approach is to carefully analyze the protein structure and consider the structural and functional characteristics of the domains. This analysis can help to identify regions that are likely to be stable and have distinct boundaries, which can be treated as separate domains. It is also important to consider any available experimental data, such as domain annotations or functional studies, to guide the domain selection process. In addition, utilizing computational tools and algorithms specifically designed for domain prediction can be helpful. These tools can analyze the protein sequence and predict potential domain boundaries based on various features, such as secondary structure, solvent accessibility and evolutionary conservation. In our work, we recommend a method that takes inspiration from classification of target EUs in CASP14 to segment the predicted models into domains. Target domains can be defined initially using *DomainParser* (Xu *et al.*, 2000 ▸), *DDOMAIN* (Zhou *et al.*, 2007 ▸) or *Sword* (Postic *et al.*, 2017 ▸) packages. Then, considering the possible differences between the predicted model and the actual crystallized portion, it will be determined whether the domain models in the terminal should be removed because of the flexibility. The remaining compact domain models will be used as the starting point for structure determination.

Compared with the first two kinds of models, the single-domain model can minimize the influence of model bias on structure construction. Therefore, for situations where the data resolution is high or the high-accuracy structure is mandatory, the single-domain model can be preferred as the starting model. However, since a complete protein can be divided into different single domains, how to choose the most suitable one also has the same problems we mentioned above. Fortunately, with the aid of *IPCAS*, a smaller model can be tolerated as the starting model, which provides more possibilities for single-domain selection (Table 4 ▸).

### 4.2. The characteristics of more challenging cases

According to the results in Tables 2 ▸–4 ▸ ▸, for the more challenging cases, such as multi-helical structures (crystal 1, PDB entry 6poo), multimeric structures (crystal 5, PDB entry 6px4; crystal 10, PDB entry 7m5f) and extremely large structures (crystal 2, PDB entry 6vr4), different characteristics are shown.

For multi-helical structures, this type of structure is the most challenging to solve with the *AlphaFold* models. The problem is twofold. Firstly, the subtle bends and kinks in the helices are more elusive which have long-range effects in the fit of the model to the target. Secondly, coiled coils induce modulations in the diffraction data which confound the maximum-likelihood targets in MR. These are the reasons for the failure of MR in case 1. These problem can be resolved by decomposing the predicted model into single-domain models, as demonstrated in cases 16, 22 and 23. We recommend the strategy of decomposing the predicted model into multiple single-domain models, as demonstrated in case 16. This approach enhances the likelihood of successful MR and facilitates rapid convergence during subsequent completion in *IPCAS*.

For multimeric structures, in addition to the accuracy of individual chain structures, the distances between chains need to be considered. Currently, *AlphaFold-Multimer* is capable of accurately predicting multimeric structures, shown in cases 6 and 12. In our test cases (cases 5, 6, 11, 12, 19, 21, 27, 28, 34 and 35), the success of MR and the subsequent rapid convergence during model completion in *IPCAS* were achieved regardless of the strategy employed. This also highlights the universality of our program and strategy. For this type of structure, the strategy with any available predicted model could be applied.

For extremely large structures, structure prediction can be challenging in terms of length and accuracy. On one hand, predicting protein structures with over 1000 amino acids requires high hardware specifications. On the other hand, local deviations in predicted models can impact the overall structure, especially for extremely large structures. For this type of structure, the strategy of predicting single-domain models in segments is recommended, as demonstrated in case 17. In comparison with case 2, although there were misplacements of single-domain models in the MR process of case 17, model completion through *IPCAS* using direct methods was able to correct the biases introduced by the MR model.

### 4.3. Contribution of *IPCAS* in phase optimization and structure completeness

Regardless of the prediction structures used as the starting point for MR, there are often deviations or incompleteness between the MR models and the reference structures. In our study, we employed direct-methods-aided model completion in *IPCAS* to correct and extend the predicted model after MR. An important characteristic of *IPCAS 2.0* is its incorporation of direct methods (Fan & Gu, 1985 ▸ *b*) into the phasing and model-building dual-space iteration, a resolution-screening method for NCS searching and an alternate model-building protocol.

**Figure 5:**
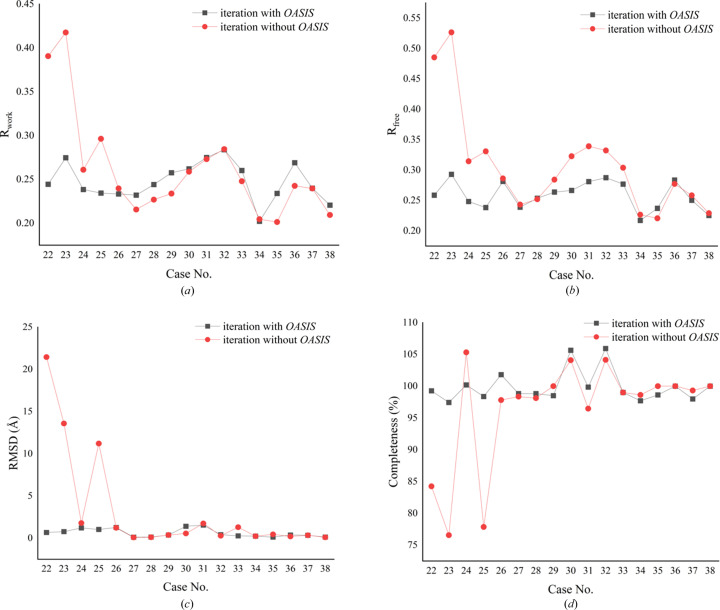
Comparison of iterative results with and without *OASIS*. (*a*) Comparison of *R*_work_. (*b*) Comparison of *R*_free_. (*c*) Comparison of RMSD. (*d*) Comparison of completeness.

**Figure 6:**
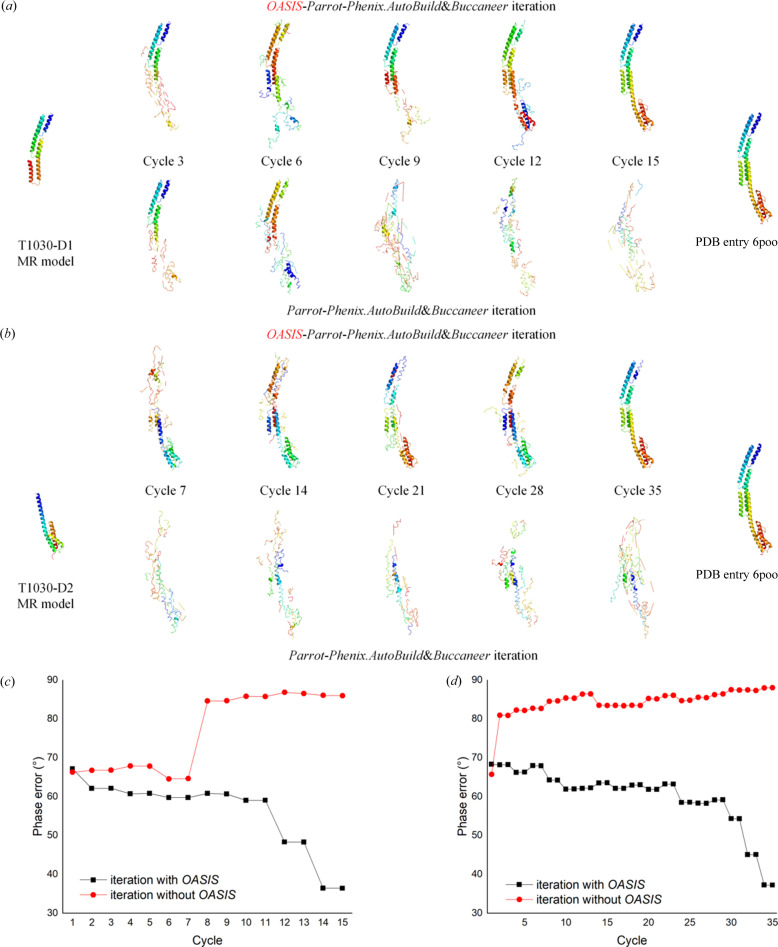
Model completion of the N-terminal helical domain of BibA with the MR model. (*a*) Model completion with the T1030-D1 MR model. Upper row, models from 15 cycles of *OASIS*–*Parrot*–*Phenix.AutoBuild*&*Buccaneer* iteration; lower row, models from 15 cycles of the *Parrot*–*Phenix.AutoBuild*&*Buccaneer* iteration (bypassing *OASIS* in the flowchart in Fig. 1 ▸). The starting models and reference PDB structures are shown on the left and right, respectively. (*b*) Model completion with the T1030-D2 MR model. Upper row, models from 35 cycles of the *OASIS*–*Parrot*–*Phenix.AutoBuild*&­*Buccaneer* iteration; lower row, models from 35 cycles of the *Parrot*–*Phenix.AutoBuild*&*Buccaneer* iteration (bypassing *OASIS* in the flowchart in Fig. 1 ▸). The starting models and reference PDB structures are shown on the left and right, respectively. (*c*) Variation of phase error during model completion of the N-terminal helical domain of BibA with the T1030-D1 MR model. (*d*) Variation of phase error during model completion of the N-terminal helical domain of BibA with the T1030-D2 MR model.

The direct-methods program *OASIS* plays a crucial role by performing a 180° phase flip of inaccurate phases in reciprocal space. Numerous publications have demonstrated the suitability of this pipeline for phase refinement and optimization of low-resolution diffraction data, even at resolutions as low as 5 Å (Fan *et al.*, 1998 ▸, 2014 ▸; Wu *et al.*, 2009 ▸; Yao *et al.*, 2014 ▸; Ding *et al.*, 2020 ▸). In our study, we tested 38 cases, and only two were unresolved due to the failure of MR. Among the remaining cases, 34 achieved phase optimization within the first five cycles of iteration, followed by structural refinement in the subsequent ten cycles. For two special cases, 22 and 23, direct-methods phase optimization required iterations until the 11th and 30th cycles, respectively, to achieve significant results. Furthermore, to demonstrate the effectiveness of *OASIS*, we tested the iteration with and without *OASIS* for the most challenging cases using the individual single-domain predicted model as the starting point, as presented in Fig. 5 ▸. It is evident that cases 22 and 23 were unable to be resolved successfully without the aid of *OASIS*. The iterations without *OASIS* failed directly and were completely destroyed within a few cycles, resulting in a set of atoms without any secondary structure, as shown in Figs. 6 ▸(*a*) and 6 ▸(*b*). Figs. 6 ▸(*c*) and 6 ▸(*d*) also show that the phase error of the iterations with the direct-methods program *OASIS* gradually decreases, whereas the iterations without *OASIS* lead to an increasing phase discrepancy, further highlighting its significance.

Moreover, the resolution screening method for non-crystallographic symmetry searching also works well in the cases with NCS copies, particularly in cases where the MR method fails to accurately determine the correct position of all models (*e.g.* case 26). In addition, by implementing an alternate model-building method (such as *Phenix.AutoBuild* and *Buccaneer*) the premature convergence of the iterative partial-structure extension process can be effectively avoided. As shown in case 31, the phase error rapidly decreases to a range approaching convergence, after two significant increases in the alternate model-building cycle.

### 4.4. RMSD analysis of the *IPCAS* models and the predicted models

**Figure 7:**
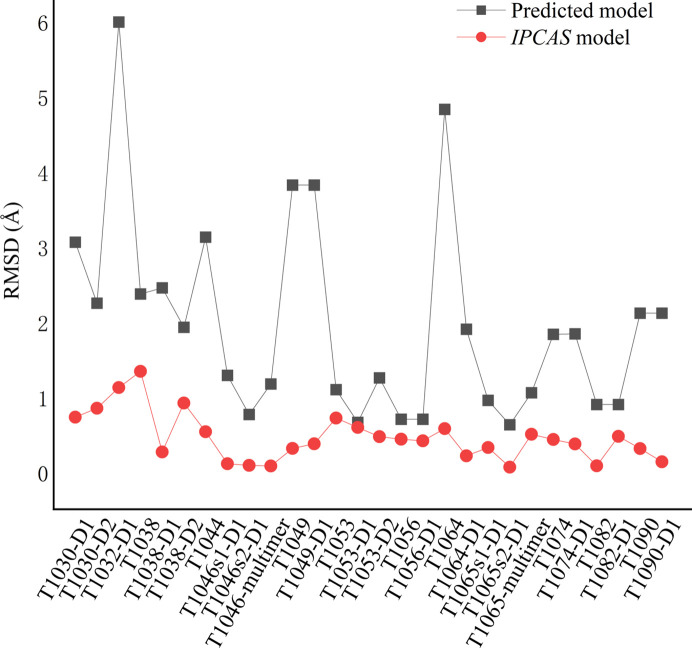
Comparison of the *IPCAS* models with the predicted models in RMSD. The RMSD values were calculated by comparison with the corresponding target PDB structures. The sequence regions evaluated by the *IPCAS* models align with the predicted models for assessment.

The RMSD analysis of the predicted models and the final structures obtained through the protocol is shown in Fig. 7 ▸. For the predicted sequence regions, the RMSD of the *IPCAS* model is consistently lower than the predicted model for all cases. This indicates that our process is capable of effectively correcting biases in predicted models, aided by the diffraction data.

### 4.5. Correction of the excess portions of the predicted models

**Figure 8:**
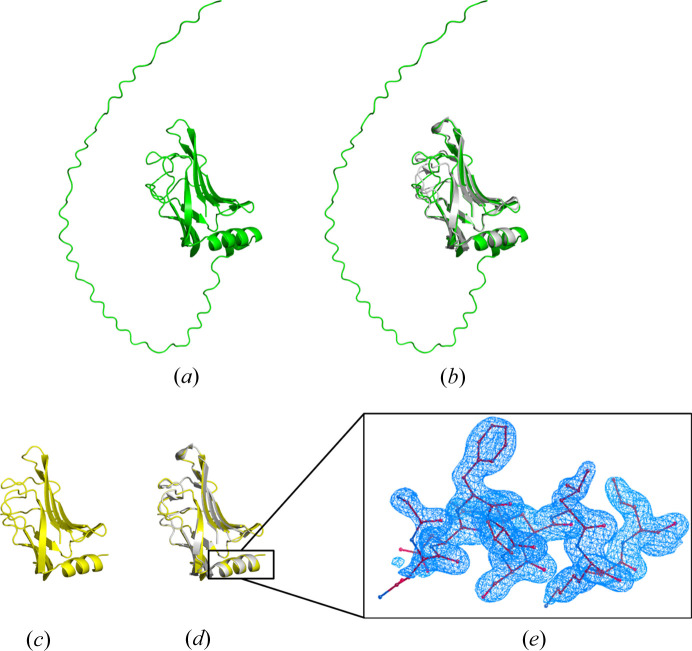
Crystal 11 (PDB entry 7oc9), target T1074. (*a*) Model from *Phaser* based on T1074. (*b*) Model (*a*) superimposed with the crystal structure. (*c*) Model from *IPCAS* based on (*a*). (*d*) Model (*c*) superimposed with the crystal structure. (*e*) The 2*F*_o_ − *F*_c_ electron density map at a contour level of 1.1σ, with a focus on the N-terminal helix region.

In the Results, particularly in Section 3.1, we observed that some of the MR models exhibit completeness greater than 100%. This phenomenon can be attributed to variations in the size of the predicted models compared with the actual regions crystallized. In such cases, it is necessary to correct the excess portions of the predicted models. For instance, in case 13, an additional section of a random loop consisting of approximately 65 residues was present at the N-terminal of the predicted model, leading to a model completeness of 151.9%. Through the direct-methods-aided model completion, the majority of the random coil region can be automatically removed. As a result, the final model obtained contains one residue more than the corresponding model in the PDB. Further details regarding this process can be found in Fig. 8 ▸.

In cases where the completeness of the final models exceeds 100%, there are two possible scenarios. Firstly, as previously discussed, it is possible that the predicted models are larger than the actual models, causing the inclusion of poorly density-resolved residues during the model completion process. Secondly, in certain cases, the modeling process involves extending partial structures to complete structures while preserving flexible regions, which lead to a final completeness greater than 100%. These flexible regions may be removed during subsequent inspection of the PDB model. This scenario can be observed in case 30.

### 4.6. The refinement and model-building process for the molecular replacement model

Once the initial models are obtained through MR, the subsequent critical steps involve refinement and model building, typically performed using software like *Phenix* (*Phenix.AutoBuild*) and *CCP4* (*Buccaneer*). Table S1 and Fig. S1 of the supporting information present the statistical results of modeling and refinement for the MR models, using *Phenix* (*Phenix.AutoBuild*), *CCP4* (*Buccaneer*) and *IPCAS*. Among the 38 cases, using an *R*_work_/*R*_free_ threshold of less than 0.30, there are 36 *IPCAS* models, 13 *AutoBuild* models and 8 *Buccaneer* models. If the *R*_free_ threshold is relaxed to 0.35, there are 36 *IPCAS* models, 21 *AutoBuild* models and 16 *Buccaneer* models [Figs. S1(*a*) and S1(*b*)]. Similar results can also be found in the curve of RMSD and completeness [Figs. S1(*c*) and S1(*d*)]. This fully demonstrates that when the initial model proves to be sufficiently accurate, it allows for the automatic generation of a nearly complete model without further manual intervention. And for the more challenging cases, such as multi-helical structures (crystal 1, PDB entry 6poo, cases 16, 22 and 23), multimeric structures (crystal 5, PDB entry 6px4, cases 5, 6, 19, 27 and 28; crystal 10, PDB entry 7m5f, cases 11, 12, 21, 34 and 35) and extremely large structures (crystal 2, PDB entry 6vr4, cases 2 and 17), *IPCAS* consistently produced the final structure, whereas only partial cases were resolved using *Phenix.AutoBuild* or *Buccaneer*.

In summary, the combination of structure prediction with MR, model building and refinement is an effective approach for biomacromolecular structure determination. And as for the *IPCAS* pipeline, the strategy is advantageous with the direct methods *P*_+_ formula for phase optimization and alternating between *Phenix.AutoBuild* and *Buccaneer* for model building to avoid local extrema, which can effectively improve the success rate of challenging cases.

## 5. Conclusions

Growing studies indicate that the prediction structure has become more accurate and reliable, and the models generated by structure prediction will accelerate the experimental determination of three-dimensional structures by improving the starting models of MR. Here, we use direct-methods-aided model completion in *IPCAS* to correct and extend the predicted model after MR. In this paper, 38 cases were tested, based on the obtained results, and several important conclusions can be drawn.

First, for a medium-sized structure, the full-length prediction structure is an ideal starting model for MR. In most cases, combined with the direct-methods-based pipeline *IPCAS*, the structure determination process could be completed automatically. Second, in cases where the full-length predicted model presents challenges, such as a single molecule with a large number of residues, an alternative strategy could be employed. By splitting and extracting the conserved structural domains from the predicted model (Kinch *et al.*, 2021 ▸), it is possible to focus on the relevant regions for MR and structure determination. Third, when dealing with much more challenging special cases, such as many significant flexibility regions exhibited in the target molecule, it may be beneficial to start with the most conservative single domain in MR and use the direct-methods-aided model extension method for the final structure determination. However, note that certain circumstances may hinder the success of this approach, such as significant deviations in the whole predicted model, crystal packing conflicts resulting in the crystallization of only a portion of the structure, or challenges in predicting multi-helical structures.

The strategies mentioned in this paper, such as prediction model searching and structural domain segmentation will be integrated as basic features in the upcoming *IPCAS 3.0* release (a webserver pipeline). We hope that our new procedure may provide an option for solving protein structures, especially for difficult cases.

## Supplementary Material

Supplementary Table S1 and Figure S1. DOI: 10.1107/S2052252523010291/lz5066sup1.pdf

## Figures and Tables

**Table 1:** The 13 crystal structures included in CASP14 and the predicted targets associated with each structure

Crystal No.	PDB entry	Resolution	No. of residues in the AU	No. of chains/unique chains	CASP EUs†	Local *AlphaFold* ‡	Residue	Reference
1	6poo	3.03	273	1/1	T1030		273	Manne *et al.* (2020 ▸)
					T1030-D1		154	
					T1030-D2		119	
2	6vr4	3.5	4332	2/1	T1031-D1		95	Drobysheva *et al.* (2021 ▸)
					T1033-D1		100	
					T1035-D1		102	
					T1037-D1		404	
					T1039-D1		161	
					T1040-D1		130	
					T1041-D1		242	
					T1042-D1		276	
					T1043-D1		148	
						T1044	2180	
3	6n64	3.3	1071	6/1	T1032		284	Chen *et al.* (2020 ▸)
					T1032-D1		170	
4	6ya2	2.5	551	3/1	T1038		199	Bahat *et al.* (2020 ▸)
					T1038-D1		114	
					T1038-D2		76	
5	6px4	1.65	427	4/2	T1046s1		74	Krieger *et al.* (2020 ▸)
					T1046s1-D1		72	
					T1046s2		142	
					T1046s2-D1		140	
						T1046s1+T1046s2	216	
6	6y4f	1.75	134	1/1	T1049		141	Jiang *et al.* (2020 ▸)
					T1049-D1		134	
7	7m7a	3.2	2125	4/1	T1053		580	Hsieh *et al.* (2021 ▸)
					T1053-D1		405	
					T1053-D2		171	
8	6yj1	2.3	338	2/1	T1056		186	To be published
					T1056-D1		169	
9	7jtl	2.04	203	2/1	T1064		106	Flower *et al.* (2021 ▸)
					T1064-D1		92	
10	7m5f	1.59	217	2/2	T1065s1		127	To be published
					T1065s1-D1		119	
					T1065s2		98	
					T1065s2-D1		98	
						T1065s1+T1065s2	225	
11	7oc9	1.5	133	1/1	T1074		202	Alexander *et al.* (2021 ▸)
					T1074-D1		132	
12	6x6o	1.52	149	2/1	T1082		97	Shi *et al.* (2020 ▸)
					T1082-D1		75	
13	7k7w	1.77	189	1/1	T1090		193	Newman *et al.* (2020 ▸)
					T1090-D1		191	

† EUs in CASP14. The suffixes ‘-D1’ and ‘-D2’ represent that the structure is divided into ‘domains’ from the predicted structure of the full sequence. The suffixes ‘-s1’ and ‘-s2’ signify that the structure is a subunit whose whole structure has two unique chains.

‡ Predicted models from local installation of *AlphaFold*. The sign ‘+’ represents that the structure is predicted by the multimer edition.

**Table 2:** Results of full-length models

Crystal No.	PDB entry	Case No.	Prediction	Prediction residue/completeness (%)	Prediction RMSD (Å)/atom	MR model TFZ	MR model *R*_work_/*R*_free_	MR model residue/completeness (%)	MR model RMSD (Å)/atom	*IPCAS* model *R*_work_/*R*_free_	*IPCAS* model residue/completeness (%)	*IPCAS* model RMSD (Å)/atom	Best cycle of iteration
1	6poo	1†	T1030	273/100.00	4.13/273	No solution							
2	6vr4	2	T1044	2180/50.32	3.12/2166	41.1	0.48/0.49	4360/100.65	3.04/4332	0.22/0.27	3954/91.27	0.53/3850	11
3	6n64	3†	T1032	284/27.52	5.99/173	5.7	0.56/0.55	1420/132.59	39.03/712	0.42/0.48	1106/103.27	23.93/134	7
4	6ya2	4	T1038	199/36.12	2.37/190	19.5	0.46/0.45	597/108.35	2.07/551	0.25/0.27	529/96.01	1.28/521	5
5	6px4	5	T1046s1	74/17.33	1.28/72	32.4	0.45/0.46	432/101.17	0.96/427	0.21/0.24	423/99.06	0.09/422	11
			T1046s2	142/33.26	0.81/142								
		6	Multimer	216/50.59	1.17/214	30.4	0.49/0.51	432/101.17	1.13/427	0.21/0.24	420/98.36	0.09/419	11
6	6y4f	7	T1049	141/105.22	3.82/134	20.4	0.52/0.52	141/105.22	3.82/134	0.23/0.26	135/100.75	0.31/134	5
7	7m7a	8	T1053	580/27.29	1.093/538	53.0	0.42/0.41	2320/109.18	1.10/2125	0.28/0.30	2230/104.94	0.80/2014	5
8	6yj1	9	T1056	186/55.03	0.70/169	9.9	0.44/0.44	372/110.06	0.76/338	0.28/0.29	358/105.92	0.44/338	8
9	7jtl	10	T1064	106/52.22	4.83/102	11.0	0.50/0.50	212/104.43	4.07/202	0.24/0.29	210/103.45	0.49/199	9
10	7m5f	11	T1065s1	127/58.53	0.95/119	41.5	0.46/0.46	225/103.69	0.83/217	0.20/0.21	218/100.46	0.24/214	3
			T1065s2	98/45.16	0.63/98								
		12	Multimer	225/103.69	1.05/217	20.6	0.52/0.50	225/103.69	1.05/217	0.21/0.22	219/100.92	0.50/214	5
11	7oc9	13	T1074	202/151.88	1.83/133	14.3	0.47/0.47	202/151.88	1.82/133	0.26/0.27	134/100.75	0.43/133	14
12	6x6o	14	T1082	97/65.10	0.89/75	12.7	0.53/0.52	97/65.10	0.85/73	0.23/0.25	146/97.99	0.08/146	13
13	7k7w	15	T1090	193/102.12	2.11/189	19.4	0.49/0.49	193/102.12	2.13/189	0.21/0.24	192/101.59	0.31/189	5

† No solution.

**Table 3:** Results of the multiple single-domain models

Crystal No.	PDB entry	Case No.	Prediction	Prediction residue/completeness (%)	Prediction RMSD (Å)/atom	MR model TFZ	MR model *R*_work_/*R*_free_	MR model residue/completeness (%)	MR model RMSD (Å)/atom	*IPCAS* model *R*_work_/*R*_free_	*IPCAS* model residue/completeness (%)	*IPCAS* model RMSD (Å)/atom	Best cycle of iteration
1	6poo	16	T1030-D1	154/56.41	3.07/154	13.3	0.47/0.48	273/100.0	3.24/273	0.23/0.28	271/99.27	0.79/271	15
			T1030-D2	119/43.59	2.24/119								
2	6vr4	17	T1031-D1	95/2.19	2.99/95	6.8	0.51/0.52	3316/76.55	49.82/3204	0.21/0.26	4051/93.51	2.43/4018	14
			T1033-D1	100/2.31	1.59/100								
			T1035-D1	102/2.35	0.82/102								
			T1037-D1	404/9.33	1.60/373								
			T1039-D1	161/3.72	3.03/161								
			T1040-D1	130/3.00	3.56/130								
			T1041-D1	242/5.59	1.76/242								
			T1042-D1	276/6.37	1.64/253								
			T1043-D1	148/3.42	2.52/148								
4	6ya2	18	T1038-D1	114/20.69	2.45/114	20.5	0.41/0.42	570/103.45	10.73/551	0.24/0.29	532/96.55	1.36/524	9
			T1038-D2	76/13.79	1.92/76								
5	6px4	19	T1046s1-D1	72/16.86	1.28/72	34.4	0.45/0.46	424/99.3	0.94/424	0.22/0.25	425/99.53	0.11/423	7
			T1046s2-D1	140/32.79	0.76/140								
7	7m7a	20	T1053-D1	405/19.06	0.66/359	35.1	0.40/0.40	2304/108.42	0.94/2123	0.27/0.28	2268/106.73	0.67/2098	3
			T1053-D2	171/8.05	1.25/170								
10	7m5f	21	T1065s1-D1	119/54.84	0.95/119	43.5	0.46/0.47	217/100.0	0.83/217	0.20/0.22	216/99.54	0.24/212	3
			T1065s2-D1	98/45.16	0.63/98								

**Table 4:** Results of individual single-domain models

Crystal No.	PDB entry	Case No.	Prediction	Prediction residue/completeness (%)	Prediction RMSD (Å)/atom	MR model TFZ	MR model *R*_work_/*R*_free_	MR model residue/completeness (%)	MR model RMSD (Å)/atom	*IPCAS* model *R*_work_/*R*_free_	*IPCAS* model residue/completeness (%)	*IPCAS* model RMSD (Å)/atom	Best cycle of iteration
1	6poo	22	T1030-D1	154/56.41	3.06/154	6.5	0.53/0.52	154/56.41	3.39/154	0.24/0.26	271/99.27	0.67/271	14
		23	T1030-D2	119/43.59	2.24/119	8.6	0.51/0.53	119/43.59	2.31/119	0.27/0.29	266/97.44	0.77/266	35
3	6n64	24	T1032-D1	170/15.87	5.69/170	7.1	0.52/0.52	1020/95.24	7.40/1020	0.24/0.25	1073/100.19	1.21/1035	10
4	6ya2	25	T1038-D1	114/20.69	2.45/114	18.2	0.48/0.45	342/62.07	2.06/324	0.23/0.24	542/98.37	1.03/536	10
		26	T1038-D2	76/13.79	1.92/76	10.3	0.52/0.51	228/41.38	21.03/227	0.23/0.28	561/101.81	1.27/547	10
5	6px4	27	T1046s1-D1	72/16.86	1.28/72	19.3	0.52/0.52	144/33.72	1.27/144	0.23/0.24	422/98.83	0.11/422	9
		28	T1046s2-D1	141/32.79	0.76/140	31.1	0.49/0.49	280/65.57	0.72/280	0.24/0.25	422/98.83	0.13/414	3
6	6y4f	29	T1049-D1	134/100.00	3.82/134	20.3	0.51/0.52	134/100.0	3.81/134	0.26/0.26	132/98.51	0.37/132	3
7	7m7a	30	T1053-D1	405/19.06	0.66/359	33.9	0.47/0.47	1620/76.24	0.73/1443	0.26/0.27	2245/105.65	1.41/2052	14
		31	T1053-D2	171/8.05	1.25/170	25.0	0.52/0.53	684/32.19	1.28/680	0.27/0.28	2122/99.86	1.54/1982	10
8	6yj1	32	T1056-D1	169/50.00	0.70/169	10.5	0.43/0.43	338/100.0	0.75/338	0.28/0.29	358/105.92	0.41/338	4
9	7jtl	33	T1064-D1	92/45.32	1.90/92	14.7	0.49/0.50	184/90.64	1.84/183	0.26/0.28	201/99.01	0.27/196	5
10	7m5f	34	T1065s1-D1	119/54.84	0.95/119	23.0	0.52/0.52	119/54.84	0.95/119	0.20/0.22	212/97.7	0.24/212	3
		35	T1065s2-D1	98/45.16	0.63/98	22.2	0.52/0.52	98/45.16	0.65/98	0.23/0.24	214/98.62	0.12/214	5
11	7oc9	36	T1074-D1	132/99.25	1.84/132	21.6	0.48/0.47	132/99.25	1.81/132	0.27/0.28	133/100.0	0.37/132	11
12	6x6o	37	T1082-D1	75/50.34	0.89/75	28.8	0.48/0.48	150/100.67	0.88/148	0.24/0.25	146/97.99	0.34/145	3
13	7k7w	38	T1090-D1	191/101.06	2.11/189	20.2	0.49/0.49	191/101.06	2.14/189	0.22/0.23	189/100.0	0.13/187	9
