# Supplementary material for: Solving protein structures by combining structure prediction, molecular replacement and direct-methods-aided model completion
Source: IUCrJ. 2024 Jan 13;11(Pt 2):152–67. doi: 10.1107/S2052252523010291 (PMC10916285; doi:10.1107/S2052252523010291)

IUCrJ

Volume 11 (2024)

Supporting information for article:

Solving protein structures by the combination of structure prediction, molecular replacement, and direct-method-aided model completion

Zengru Li, Haifu Fan and Wei Ding

Table S1 Results of model completion of molecular replacement model in different ways.

Crystal No.	PDB entry	Case No.	Prediction	*IPCAS* model R_{work}/R_{free}	*IPCAS* model Residue/ Completeness	*IPCAS* model RMSD(Å)/ Atom	*AutoBuild* model R_{work}/R_{free}	*AutoBuild* model Residue/ Completeness	*AutoBuild* model RMSD(Å)/ Atom	*Buccaneer* model R_{work}/R_{free}	*Buccaneer* model Residue/ Completeness	*Buccaneer* model RMSD(Å)/ Atom
1	6poo	1	T1030	no solution								
2	6vr4	2	T1044	0.22/0.27	3954/91.27%	0.53/3850	0.24/0.31	4360/100.65%	3.95/4332	0.25/0.36	4163/96.10%	2.87/4135
3	6n64	3	T1032	0.42/0.48	1106/103.27%	23.93/134	0.45/0.52	890/83.10%	33.39/324	0.44/0.59	968/90.38%	32.18/749
4	6ya2	4	T1038	0.25/0.27	529/96.01%	1.28/521	0.25/0.31	597/108.35%	1.76/551	0.26/0.34	556/100.91%	1.82/527
5	6px4	5	T1046s1<br>T1046s2	0.21/0.24	423/99.06%	0.09/422	0.21/0.24	432/101.17%	0.74/427	0.24/0.26	432/101.17%	0.09/427
		6	Multimer	0.21/0.24	420/98.36%	0.09/419	0.22/0.25	432/101.17%	1.00/427	0.24/0.28	427/100.00%	0.09/422
6	6y4f	7	T1049	0.23/0.26	135/100.75%	0.31/134	0.25/0.31	141/105.22%	3.80/134	0.32/0.36	141/105.22%	3.89/134
7	7m7a	8	T1053	0.28/0.30	2230/104.94%	0.80/2014	0.23/0.30	2320/109.18%	0.69/2125	0.26/0.37	2333/109.79%	0.92/2124
8	6yj1	9	T1056	0.28/0.29	358/105.92%	0.44/338	0.27/0.33	372/110.06%	0.40/338	0.29/0.34	370/109.47%	0.54/336
9	7jtl	10	T1064	0.24/0.29	210/103.45%	0.49/199	0.27/0.31	212/104.43%	3.81/202	0.38/0.48	209/102.96%	4.70/190
10	7m5f	11	T1065s1<br>T1065s2	0.20/0.21	218/100.46%	0.24/214	0.18/0.20	225/103.69%	0.28/217	0.23/0.25	227/104.61%	0.30/217
		12	Multimer	0.21/0.22	219/100.92%	0.50/214	0.18/0.20	225/103.69%	0.32/217	0.25/0.27	221/101.84%	0.56/217
11	7oc9	13	T1074	0.26/0.27	134/100.75%	0.43/133	0.26/0.29	122/91.73%	1.29/107	0.30/0.33	134/100.75%	1.99/133
12	6x6o	14	T1082	0.23/0.25	146/97.99%	0.08/146	0.46/0.48	97/65.10%	0.79/73	0.46/0.51	87/58.39%	0.52/73
13	7k7w	15	T1090	0.21/0.24	192/101.59%	0.31/189	0.24/0.27	193/102.12%	1.84/189	0.27/0.31	200/105.82%	0.49/183
1	6poo	16	T1030-D1<br>T1030-D2	0.23/0.28	271/99.27%	0.79/271	0.31/0.39	273/100.00%	3.26/273	0.29/0.42	259/94.87%	2.16/258
2	6vr4	17	T1031-D1<br>T1033-D1<br>T1035-D1<br>T1037-D1<br>T1039-D1<br>T1040-D1<br>T1041-D1<br>T1042-D1<br>T1043-D1	0.21/0.26	4051/93.51%	2.43/4018	0.42/0.51	3316/76.55%	37.30/2820	0.37/0.52	3059/70.61%	30.89/2616
4	6ya2	18	T1038-D1<br>T1038-D2	0.24/0.29	532/96.55%	1.36/524	0.31/0.37	570/103.45%	7.77/476	0.24/0.31	555/100.73%	1.39/544
5	6px4	19	T1046s1-D1<br>T1046s2-D1	0.22/0.25	425/99.53%	0.11/423	0.21/0.24	424/99.30%	0.72/424	0.24/0.27	432/101.17%	0.31/427
7	7m7a	20	T1053-D1<br>T1053-D2	0.27/0.28	2268/106.73%	0.67/2098	0.24/0.31	2304/108.42%	0.72/2123	0.25/0.38	2308/108.61%	0.84/2100

10	7m5f	21	T1065s1-D1<br>T1065s2-D1	0.20/0.22	216/99.54%	0.24/212	0.18/0.19	217/100.00%	0.33/217	0.23/0.25	219/100.92%	0.29/217
1	6poo	22	T1030-D1	0.24/0.26	271/99.27%	0.67/271	0.42/0.52	154/56.41%	3.16/154	0.38/0.52	167/61.17%	14.43/150
		23	T1030-D2	0.27/0.29	266/97.44%	0.77/266	0.44/0.52	119/43.59%	2.42/119	0.40/0.53	122/44.69%	2.19/106
3	6n64	24	T1032-D1	0.24/0.25	1073/100.19%	1.21/1035	0.38/0.46	1020/95.24%	7.60/1020	0.39/0.51	1002/93.56%	7.29/937
4	6ya2	25	T1038-D1	0.23/0.24	542/98.37%	1.03/536	0.34/0.39	342/62.07%	1.42/324	0.33/0.40	377/68.42%	2.07/343
		26	T1038-D2	0.23/0.28	561/101.81%	1.27/547	0.47/0.51	152/27.59%	1.83/152	0.44/0.54	189/34.30%	13.43/184
5	6px4	27	T1046s1-D1	0.23/0.24	422/98.83%	0.11/422	0.43/0.45	144/33.72%	1.12/144	0.46/0.50	148/34.66%	1.23/144
		28	T1046s2-D1	0.24/0.25	422/98.83%	0.13/414	0.31/0.34	280/65.57%	0.15/280	0.25/0.27	427/100.00%	0.13/422
6	6y4f	29	T1049-D1	0.26/0.26	132/98.51%	0.37/132	0.23/0.27	134/100.00%	3.72/134	0.31/0.36	127/94.78%	0.88/125
7	7m7a	30	T1053-D1	0.26/0.27	2245/105.65%	1.41/2052	0.39/0.46	1620/76.24%	0.66/1443	0.33/0.48	1921/90.40%	8.08/1576
		31	T1053-D2	0.27/0.28	2122/99.86%	1.54/1982	0.46/0.50	684/32.19%	1.15/680	0.41/0.56	898/42.26%	18.58/796
8	6yj1	32	T1056-D1	0.28/0.29	358/105.92%	0.41/338	0.27/0.33	338/100.00%	0.38/338	0.28/0.34	351/103.85%	0.48/336
9	7jtl	33	T1064-D1	0.26/0.28	201/99.01%	0.27/196	0.29/0.33	184/90.64%	1.27/183	0.38/0.48	184/90.64%	17.22/153
10	7m5f	34	T1065s1-D1	0.20/0.22	212/97.70%	0.24/212	0.34/0.37	119/54.84%	0.40/119	0.40/0.43	119/54.84%	0.61/117
		35	T1065s2-D1	0.23/0.24	214/98.62%	0.12/214	0.36/0.40	98/45.16%	0.16/98	0.31/0.35	182/83.87%	1.11/174
11	7oc9	36	T1074-D1	0.27/0.28	133/100.00%	0.37/132	0.23/0.25	132/99.25%	1.29/132	0.30/0.33	134/100.75%	1.60/133
12	6x6o	37	T1082-D1	0.24/0.25	146/97.99%	0.34/145	0.23/0.25	150/100.67%	0.31/148	0.27/0.30	152/102.01%	0.67/148
13	7k7w	38	T1090-D1	0.22/0.23	189/100.00%	0.13/187	0.24/0.28	191/101.06%	1.84/189	0.28/0.32	179/94.71%	0.58/176

Figure S1 Comparison of model completion in different ways. (a) Comparison of R_{work}. (b) Comparison of R_{free}. (c) Comparison of RMSD. (d) Comparison of completeness.

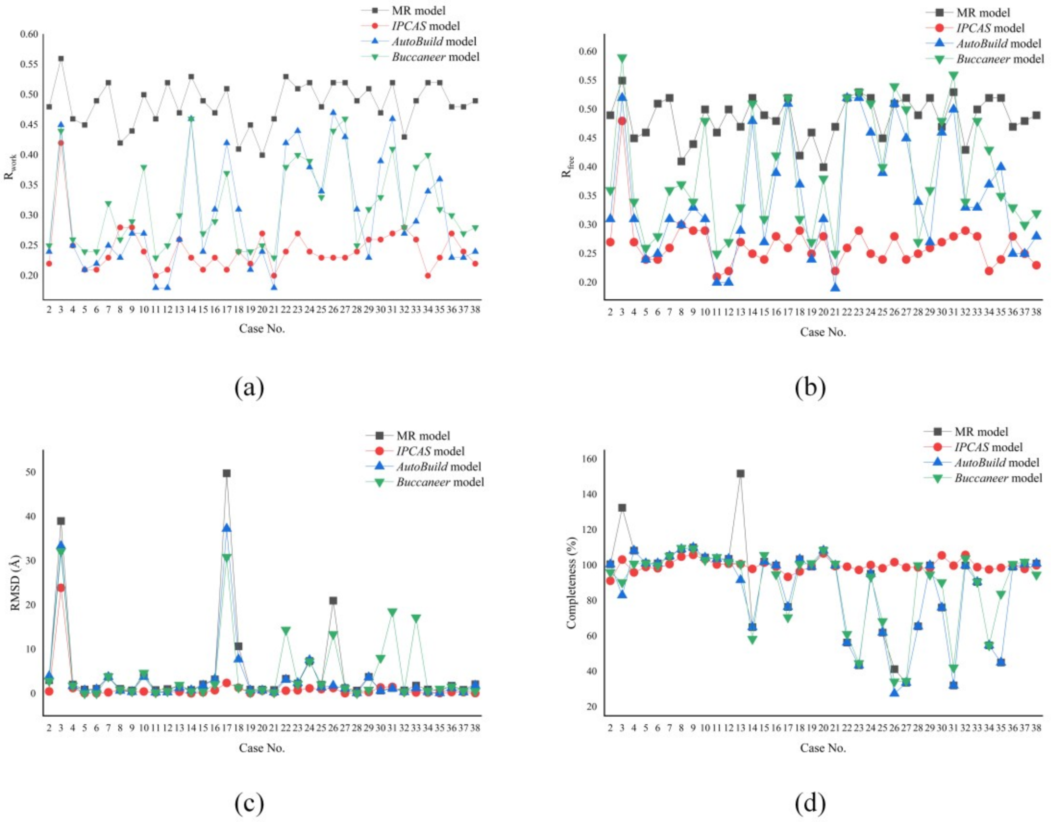

Supplement: Supplementary file 1 [file m-11-00152-sup1.pdf]
